# Editorial: Dietary patterns affecting cardiovascular health

**DOI:** 10.3389/fnut.2024.1469068

**Published:** 2024-08-19

**Authors:** Galya Bigman, Amedeo Amedei

**Affiliations:** ^1^Department of Epidemiology and Public Health, School of Medicine, University of Maryland, Baltimore, MD, United States; ^2^University of Florence, Florence, Italy

**Keywords:** cardiovascular health, cardiovascular diseases (CVDs), cardiometabolic outcomes, dietary patterns, sodium, fruits, weight loss, carbohydrate restriction

Over half a billion people globally are affected by cardiovascular diseases (CVDs), which caused 20.5 million deaths in 2021—nearly a third of all global deaths and a significant increase from previous estimates of 12.1 million deaths in 1990. Ischaemic heart disease (IHD) and stroke account for approximately 85% of all CVD deaths (1). This burden disproportionately affects low- and middle-income countries, where four out of five CVD deaths occur (1).

Poor dietary habits such as high sodium intake, low whole grain consumption, and inadequate fruit intake significantly impact cardiovascular health globally. Addressing these habits through global public health strategies, including food- and nutrient-based guidelines, is crucial for CVDs prevention and management (2). However, the specific mechanisms linking dietary components to cardiovascular function require further elucidation and recent decades have seen a shift toward assessing overall dietary patterns rather than isolated food components.

Therefore, this Research Topic, “*Dietary Patterns Affecting Cardiovascular Health*,” aims to consolidate new research on dietary patterns and their impact on cardiovascular health, especially in underrepresented populations, to deepen our understanding of diet-health interactions.

Overall, we received 62 submissions, with 40 being rejected following initial editorial assessment. Ultimately, 22 articles underwent one or more rounds of peer review, exploring various dietary factors such as dietary risks, low fruit consumption, sodium intake, lycopene, and supplements like thiamine and folic acid. The studies also investigated different dietary patterns such as carbohydrate restriction, ketogenic-like diets, and ultra-low-fat diets. Additional topics included ultra-processed foods, high-fat meals, and the effects of three diet interventions on weight loss (low carbohydrate, low fat, and low calorie). Furthermore, research covered plant-based vs. animal-based protein intake, dietary intake of live microbes, and the dietary inflammation index.

Data sources encompassed the Global Burden of Disease (GBD) 2019, national surveys from the USA, Korea, the UK Biobank and the FinnGen, as well as data from Tehran, China, and Pakistan and Belt and Road (B&R) countries. Studies employed different methodologies including observational epidemiological studies, randomized controlled trials, Mendelian randomization analyses, and reviews.

The studies explored diverse aspects of cardiovascular health (CVH) such as CVDs, IHD, stroke, myocardial infarction (MI), vascular function, cardiometabolic outcomes, heart failure (HF), abdominal aortic calcification (AAC), severe coronary artery disease (CAD), atherosclerotic cardiovascular disease (ASCVD), acute coronary syndrome (ACS), hypertension (HTN), H-type hypertension and dyslipidemia. Our summary focuses on the main dietary factors investigated in these studies as follows:

Three studies utilized the GBD 2019 data to examine the global burden of CVDs from 1990 to 2019 and the relationships with different dietary risks. Pan et al. found that the number of deaths and disability-adjusted life years (DALYs) due to a diet low in fruits increased by 31.5% and 27.4%, respectively. Among the tertiary diseases, IHD, stroke, and diabetes and kidney disease were the top three contributors to that increase, with the burden being significantly higher in the elderly. In Zhang Y. et al. although there was a significant overall reduction in stroke mortality and DALYs attributable to dietary risk across Belt and Road (B&R) countries, there were geographical disparities in age-standardized rates (ASR) for stroke mortality and DALYs, with some regions experiencing rapid declines (e.g., Estonia in Eastern Europe) while others observed increases (e.g., the Philippines). Yan et al. focused on regional and country levels across China and Pakistan and found that the all-ages CVD burden attributable to dietary risks and high BMI increased by ~2–3-fold in China and by 3–5-fold in Pakistan.

Two studies have examined sodium intake and CVDs using Mendelian randomization analysis. This approach relies on genetic variants as instrumental variables, providing more robust results compared to traditional observational studies, which have shown inconsistent outcomes. Fu et al. measured the sodium intake by the urinary sodium/creatinine ratio (UNa/UCr), which showed a significant positive relationship with seven specific CVDs types. In contrast, Yuan et al. also suggest that higher sodium intake is associated with an increased risk of HF as well as with HTN. However, they noted that excessively low sodium intake may not necessarily be beneficial, with maximum benefits observed at a sodium intake level of around 3,000 mg/day.

Three studies focused on a specific nutrient, where two administrated supplementations and one by analyzing FFQ data. Chen et al. conducted a randomized clinical trial with 1,567 Chinese adults aged ≥45 years with H-type HTN, defined as essential HTN with an increased plasma homocysteine level (≥10 μmol/L), which accounts for about 75% of HTN among Chinese patients. They showed that 0.8 mg of folic acid is the optimal dosage for balancing efficacy (increasing 5-methyl tetrahydrofolic acid [5-MTHF] and lowering homocysteine) while minimizing the undesirable elevation of unmetabolized folic acid (UMFA), which is due to excessive intake of folic acid based on previous research. Yue et al. aimed to determine the survival benefit of thiamine supplementation in critically ill patients with MI in the ICU using a retrospective cohort analysis of medical records. The results showed that thiamine supplementation significantly decreased the risk of in-hospital, 30-day, and 90-day mortality, suggesting that thiamine use might be associated with better survival outcomes in critically ill MI patients. Finally, Amjadi et al. conducted a Tehran-based case-control study using a 237-item FFQ to assess dietary lycopene intake. They analyzed data from 443 IHD patients and 443 controls, finding 33% lower odds of IHD in the highest vs. lowest quartile of lycopene intake (p = 0.036).

Five studies examined the association between different macronutrients and CVDs. Angelotti et al. analyzed a nationally representative sample of over 35,000 US individuals followed for an average of 10 years. They found that carbohydrate restriction (< 45% of energy intake) was not associated with increased or decreased mortality from all causes, CVD, or cardiometabolic disease. This held true even after stratifying the analysis by different fat types and amounts. Aronica et al. analyzed the DIETFITS trial, which compared weight loss and cardiometabolic outcomes between participants following either a ketogenic-like diet (KLD) or an ultra low-fat diet (ULF) for 3 and 12 months. Less than 10% of participants maintained strict KLD or ULF diets at 3 months. Overall, extreme dietary restriction of fat or carbohydrates led to substantial initial benefits in weight loss and improvement in insulin sensitivity, with slight advantages in diet quality and blood lipid parameters favoring KLD over ULF after 12 months despite dietary relapse.

Losavio et al. examined factors influencing weight loss success across three diet interventions (low carbohydrate, low fat, and low calorie) among obese patients. Despite similar average weight loss of−5.1 ± 4.0 kg over 12–16 weeks across all diets, significant inter-individual variation was observed in weight loss outcomes. Each diet type demonstrated unique cardiometabolic health benefits, highlighting the importance of personalized diet interventions to enhance weight loss and improve overall cardiometabolic outcomes. Jung et al. analyzed data from Korean adults aged 40 years and older to examine the association between the percentage of energy intake from ultra-processed foods (UPFs) and CVH metrics defined by the American Heart Association. It was found that individuals in the highest quartile of UPF intake had a 26% higher likelihood of having inadequate CVH compared to those in the lowest quartile. The study highlights the potential benefits of limiting UPF consumption as a preventive measure against CVDs. Lastly, Szczepańska et al. assessed dietary habits among MI patients, highlighting frequent consumption of refined carbohydrates like white bread and pasta. Despite insights from the pro-Healthy Diet Index, the study suggests potential dietary errors needing further investigation due to limitations in sample size and assessment methods.

Two studies focused on amino acids intake. Gao and Hou reviewed the recent evidence linking branched-chain amino acids (BCAAs)—leucine, isoleucine, and valine—with HF, a critical stage in cardiovascular diseases with high mortality and limited treatment options. They explore complex metabolic pathways, revealing how disrupted BCAA metabolism is associated with conditions such as hypertension, obesity, and atherosclerosis, contributing to HF progression. They discusses therapeutic strategies, the potential of modulating BCAAs metabolism to treat heart failure, and consider BCAAs and their metabolites as biomarkers for assessing cardiac metabolic risk. Chung et al. investigated the association between amino acid intake and dyslipidemia in Korean adults using data from the Ansan and Ansung Study and the Health Examinee Study. They analyzed data from over 35,000 participants initially without dyslipidemia, with an average follow-up of 5.7 years. Higher intake of essential and nonessential amino acids was associated with a reduced risk of dyslipidemia. Plant-based protein intake showed a negative association, while animal-based protein intake did not significantly affect dyslipidemia risk after adjusting for energy-adjusted fat intake. These findings suggest that amino acid intake, regardless of protein source, may have a protective effect against dyslipidemia.

Two other studies examine the potential of gut microbiota in relations with CVDs. Huo et al. explored the association between dietary intake of live microbes and AAC, using data from the National Health and Nutrition Examination Survey (NHANES). The results showed that higher intake of dietary live microbes was significantly associated with a lower risk of severe AAC and decreased AAC scores after adjusting for covariates and suggested a potential protective effect of dietary live microbes against AAC. Jiao et al. reviewed the growing evidence on the therapeutic potential of gut microbiota (GM) in addressing HTN. Using bibliometric analysis tools like CiteSpace and VOSviewer, the study identified 1,730 articles published from 2014 to 2023. The research spans 88 countries and involves 9,573 authors across 593 journals, highlighting the global interest and collaboration in this field. Key topics include GM metabolites, high-salt diet, and the impact of conditions like metabolic syndrome and chronic kidney disease on HTN.

Two studies address anti-inflammatory properties in relation with CVDs. Dadaei et al. explored the relationship between the dietary inflammation index (DII) and severe CAD. Using data from 275 adults undergoing elective angiography, DII was measured using a valid semi-quantitative 168-item food frequency questionnaire (FFQ). The study found that individuals with higher DII scores (indicating higher intake of pro-inflammatory foods) had significantly increased odds of severe CAD, hypercholesterolemia, reduced HDL-cholesterol levels, and hypertension compared to those with lower DII scores (indicating higher intake of anti-inflammatory foods). Zhang J. et al. investigated the relationship between composite dietary antioxidant index (CDAI) and estimated 10-year ASCVD risk among U.S. adults using data from the NHANES. It included 10,984 adults aged 18 years and above. The study found that higher CDAI scores, indicating greater dietary antioxidant intake, were associated with a lower 10-year ASCVD risk after adjusting for potential confounders.

Lastly, there were two studies that focused on psychological factors. So et al. assessed dietary and psychological factors in Korean patients with an ACS compared to controls. ACS patients showed higher intake of sweets and fish/seafood, higher levels of depressive symptoms, and lower life satisfaction across various domains. High sweet intake and low total life satisfaction scores independently contribute to increased risk of ACS, with a synergistic interaction further amplifying their impact on ACS development. Baynham et al. investigated how consuming a high-fat meal exacerbates the negative impact of mental stress on vascular function. The results showed that the high-fat meal significantly increased plasma triglyceride levels compared to the low-fat meal. Both groups experienced similar acute impairments in endothelial function immediately after stress, but those who consumed the high-fat meal showed prolonged impairment 90 min post-stress.

In summary, all the studies included in this Research Topic either provided insight on well-established nutrients such as sodium and folic acids to establish optimal intake, or they offered new insights on popular dietary patterns such as carbohydrate restriction, ketogenic-like diets, ultra-low-fat diets, and plant-based protein diet, along with different approaches for weight loss. Additionally, specific mechanisms linking dietary components were examined, including live microbes and the dietary inflammation index. Finally, they explored the interplay between psychological disorders, diets, and CVDs, an area that warrants further investigation. Clearly, additional research is necessary to replicate these findings and to solidify their implications.

To conclude, we would like to express our profound gratitude to “Frontiers in Nutrition” for the opportunity to serve as editors for this Research Topic. This challenging and motivating experience has been highly educational, and we look forward to continuing this endeavor. We extend our heartfelt thanks to the contributing authors for sharing their valuable research, which we believe will significantly impact clinical practice. Lastly, we are deeply appreciative of our reviewers for their time and insights, which have undoubtedly enhanced the quality of these studies.

## Author contributions

GB: Conceptualization, Writing – original draft, Writing – review & editing. AA: Writing – review & editing.

## References

[1] World Heart Report 2023: Confronting the World's Number One Killer. Geneva: World Heart Federation. (2023). Available at: https://world-heart-federation.org/wp-content/uploads/World-Heart-Report-2023.pdf (accessed July 9, 2024).

[2] GBD 2017 Diet Collaborators. Health effects of dietary risks in 195 countries, 1990-2017: a systematic analysis for the Global Burden of Disease Study 2017. Lancet. (2019) 393:1958–72. 10.1016/S0140-6736(19)30041-8 30954305 PMC6899507

